# Supplementary material for: Oral Health Promotion under the 8020 Campaign in Japan—A Systematic Review
Source: Int J Environ Res Public Health. 2023 Jan 19;20(3):1883. doi: 10.3390/ijerph20031883 (PMC9915309; doi:10.3390/ijerph20031883)
Supplement: Supplementary file 1 [file ijerph-20-01883-s001.zip › ijerph-2129254-supplementary.pdf]

Supplementary Materials

Study Selection

After duplications were removed, all articles were chosen in two steps. Step 1: Using PECO criteria, the study was chosen based on its title and abstract. Step 2: Involved reading the complete text of the chosen articles and selecting those that met the criteria for inclusion. Three reviewers (TZ, AR, and FC) searched and screened for articles written in English, while another two Japanese reviewers screened the Japanese articles (HO* and AT). Two principal investigators (ST and RK) checked the lists of included studies and excluded studies. Any disagreements between reviewers were resolved by discussion with principal investigators (ST and RK).

Data Extraction

The included articles were extracted by the following criteria: database source, year of publication, author, study design, number of subjects, population, exposure, or intervention (related to the 8020 Campaign, number of teeth, oral health conditions, etc.), control or comparison group, outcome, major findings, and funding sources (The 8020 Foundation or other funding). Our outcomes of interest are any changes in oral health, general health, or both, due to the 8020 Campaign. For oral health, the outcome included mastication(1) function and any other oral health conditions related to missing teeth. And for general health, the outcome included any systemic or general health conditions related to the number of teeth, and quality of life.(2)

Data extraction from English articles was independently performed by three reviewers (TZ, AR and FC), while extraction of data for Japanese articles was carried out by the two Japanese reviewers (HO* and AT). Any ambiguities were resolved through discussion between reviewers or consultation with one of the principal investigators (ST and RK).

Quality assessment

The evaluation criteria were adapted from National Heart, Lungs, and Blood Institutes at the National Institute of Health Evidence Quality Grading System (NIH quality assessment tool), which consists of the strength of evidence, risk of bias, consistency, directness, and precision.(3) Publication bias criteria were also evaluated.(4) 1) *Strength of evidence*, is assessed the degree of certainty based on the type of evidence and study design. There are 3 categories, each with score: high (+1), moderate (+0), and low (-1). 2) *Risk of Bias*, evaluated using the National Institutes of Health Critical Appraisal tool for cohort and cross-sectional studies.(5) This tool consists of 14 questions, which is suitable for cohort, and 10 questions that meet the criteria for cross-sectional studies. This tool covers 13 aspects of quality assessment, including clarity of the research purpose or research question; definition, selection, composition, and participation of the study population; assessment of exposure and outcome variables; exposure and outcome evaluation; time frame and follow up of study; as well as study analysis and power. Each question from the checklist gives different answers such as Yes, No, and CD (Cannot Determine)/NA (Not Applicable)/NR (Not Reported). 3) *Consistency,* which is measured by the similarity of outcomes among the included studies. The studies are considered "consistent" if the result has the same direction with at least 3 articles in our included studies. 4) *Directness*, is measured by the degree of generalizability. The article will be considered as "have directness" if the result can be generalized from a specific population to a more general population. 5) *Precision*, measured from the statistical analysis used in the study. The study was considered "precise" if it conducted a proper statistical analysis. 6) *Publication bias*, which is considered from the source of funding, journal reputation, double publication, and the language used. The last four criteria scored 0 if provide evidence and -1 for not being able to give evidence.

Risk of bias assessment

Quality assessment was performed by three reviewers (TZ, AR, and FC) for English articles and two reviewers (HO* and AT) for Japanese articles. For overall quality, reviewers combined the score from the strength of evidence score, risk of bias assessment, consistency, directness, precision, and publication bias scores. The scoring and grading system was carried out based on the consensus among the reviewers. There are 3 grades of quality results: good, fair, and

poor. The two principal investigators (ST and RK) rechecked all evaluation results. If there were any disagreements, they were handled by discussion among the reviewers.

This review includes a total of 25 articles, however, one of them has two study designs. To check the details, we conducted quality assessments separately for the article using two different study designs. Overall, based on the calculations from 6 criteria, out of 26 studies, 1 article has “good” quality ((6)), 22 articles ((7, 8, 9, 10, 11, 12, 13, 14, 15, 16, 17, 18, 19, 20, 21, 22, 23, 24, 25, 26, 27)) have “fair” quality, and 3 articles ((28, 29, 30)) have “poor” quality. According to the results of the quality assessment, most of the articles had issues with directness, accuracy, and publication bias. Since the findings from these studies cannot be extended to other populations, directness is difficult to achieve. As the 8020 Campaign is being conducted in Japan, many studies were published in local journals, and in Japanese. This makes publication bias difficult to avoid.

The included studies were mainly cross-sectional (18 studies), cohort (6 studies), interventional (1 study), and before-after (1 study) studies. One article was divided into 2 studies in data extraction table, as it reported two different studies in one article.(21) The targeted populations were mainly the older population (≥60 years of age) (20 studies), adults (4 studies), and children were included in only one article. The most common outcomes were oral health. As for the languages, 20 included studies were reported in Japanese, and remaining 5 studies were in English. The major funding sources for studies were the 8020 Promotion Foundation (5 studies),(11, 24, 25, 26, 27) and the government (5 studies),(9, 12, 13, 15, 18) private sector (1 study). (20) Remaining 14 papers did not provide details of funding source.

The results of the current review found that there was "good" quality evidence in one study on the association between having a full dentition (28 teeth) and a lower risk of mortality. There was a “fair” quality of evidence on the associations between number of teeth with masticatory function, salivary secretion, regular dental check-ups, and general health. The risk of bias, publication bias, and indirectness were all associated with an assessment of a "fair" quality. The relationship between number of teeth and occlusal force or oral flora was supported by only minimal evidence due to “poor” quality evidence. Two poor-quality articles used occlusal force as an outcome and the number of teeth as an exposure, they concluded that occlusal force was not affected if there were enough remaining teeth. These articles were assessed as having a high risk of bias because they did not recruit the same population of control or comparison groups as the intended population. There was no information on confounding variables available in these articles. Furthermore, the approach was not clearly communicated, and the structures were not appropriately developed. From our findings, it was unclear that what activities are considered as parts of the 8020 Campaign. Despite the fact the some included studies were funded by the 8020 foundation and some researchers mentioned the 8020 Campaign in their articles, there was no clear explanation concerning the direct relationship between their findings and the 8020 Campaign.

Excluded Study List

Table S1. Excluded Studies List

No.	Database	Author Name	Year	Title JPN	Title ENG	Reason of exclusion
1	Google scholar	Kazuhiro Shimoyama, Yayoi Okada, Tatsuro Uchida, et al.	1996	在宅寝たきり老人の口腔ケアに関する研究	Study on Oral Health Care for the Homebound Elderly (I) Oral Health Awareness of Public Health Nurses	1
2	Google scholar	Tetsuo Hanagata, Fumiyo Tamura, Takeshi Kikutani et al.	2009	介護老人福祉施設における口腔ケア・マネジメントの効果	Effectiveness of Oral Care Management in Long-term Care Facilities for the Elderly	1
3	Google scholar	Kanae Esashi, MasahikoKikuchi, Mitsuru Shimonishi et al.	2011	高齢歯科患者における口腔内カンジダ菌の検出に関連する要因	Factors Associated with the Detection of Oral Candida in Elderly Dental Patients.	1
4	Google scholar	Masahiro Takahashi, Mitsuo Kishi	2006	舌苔中の歯科疾患関連細菌と口腔内状況との関連性	The association of oral Disease-associated Bacteria in the Tongue Coating and Oral Conditions	1
5	Google scholar	Masao Ishikawa, Noriko Takei, Takanori Ishii et al.	2006	高齢者の口腔微生物叢に関する研究 : 70 歳者の口腔状態と口腔微生物叢	Studies on the Oral Microbiota of the Elderly : Oral Status and Oral Microbiota of 70-Year-Olds	1
6	Google scholar	Toru Ichihashi, Noriko Kumagai, Masaki Koshimizu et al.	1991	名古屋市内某事業所における歯科保健活動第 2 報	Second Report on Dental Health Activities at a Certain Office in Nagoya City	1
7	Google scholar	Kimie Fujikawa, Koji Hirose	2019	過疎高齢化地域と都市近郊地域における高齢者の口腔状態，口腔保健行動および意識の比較	A Comparison of Oral Health Status, Oral Health Behavior, and Attitudes of the Elderly in a Depopulated Aging Area and a Suburban Area	1
8	Google scholar	Masaru Matsumoto	1997	妊産婦の口腔環境変化に関する疫学的研究	Epidemiological study on changes in the oral environment of pregnant and nursing women	1
9	Google scholar	Koji Arai, Yasunori Sumi, Hiroko Miura et al.	2001	高齢者の口腔状況と機能に関する研究	Research on the oral status and function of the elderly	1
10	PubMed	Munetsugu Tashiro, SoIchiro Hirata, Kousuke Oozawa, Junko Shirai, Kentaro Taniguchi, Norio Kasahara	2020		How to Increase Number of Present Teeth in Elderly: Consideration of Percentile Curves for Present Teeth in Tokyo	1
11	EMBASE	Bandou R., Ichioka H., Kawamoto M., Ikegaya H.	2021		Periodontal status and self-reported systemic health of periodontal patients regularly visiting dental clinics in the 8020 Promotion Foundation Study of Japanese Dental Patients	1
12	EMBASE	Furuta M., Fukai K., Aida J., Shimazaki Y., Ando Y., Miyazaki H., Kambara M., Yamashita Y.	2019		How to Increase Number of Present Teeth in Elderly: Consideration of Percentile Curves for Present Teeth in Tokyo	1

No.	Database	Author Name	Year	Title JPN	Title ENG	Reason of exclusion
13	Ichushi	Sawaya TADATSU	2004	勤労者のストレス調査結果 勤労者のストレスと口腔の健康度に関する検討(1)	Result of Stress Survey for Workers : A Study of Stress and Oral Health in the Workplace (1)	1
14	Google scholar	Jin et al.,	2014	健康づくり実践高齢者の口腔内状況と健康度との関連についての検討 : 健康改善カードの活用	Examination of the relationship between oral status and health level of elderly people practicing health promotion : Use of Health Improvement Cards	1
15	Google scholar	Ikebe et al.,	1997	介護の必要な高齢者の口腔内状態と義歯使用状況　生活環境及び痴呆の有無による影響	State of Oral and Denture Wearing in Handicapped Elderly People -Influence of residence and dementia-	1
16	Ichushi	Nakayama et al.,	2011	第 3 次産業勤労者の口腔保健行動と意識　大阪府堺市西区における 2016 年度アンケート調査	Oral Health Behavior and Awareness of Tertiary Industry Workers: A 2016 Questionnaire Survey in Nishi-ku, Sakai-shi, Osaka	1
17	Ichushi	Ohi et al.,	2005	都市部住宅地域における在宅高齢者の口腔状態 鶴ヶ谷プロジェクト	Oral health status in an elderly urban population: the Tsurugaya project	1
18	Google scholar	Tetsunori OZAKI, IIiroshi MOCHIZUKI, Yumiko ICHIKAWA, et al.	1999	成人歯科保健事業に関する研究 : 事業実施方法と受診者の口腔状況について	A Study on Dental Health Services for Adults: How to Provide the Service,<br>and the Status, of Oral Health of the Examination Subjects	2
19	Google scholar	Yuichi ANDO, AkihiroYOSHIHARA, Yoshikazu SEIDA, et al.	2000	高齢者を対象とした歯科疫学調査におけるサンプルの偏りに関する研究 : 質問紙の回答状況および健診受診の有無別にみた口腔および全身健康状態の比較	The Study of Sampling Biasin an Oral Health Survey of Elderly -Comparison of Oraland Genera!Health Conditionbetween Respondents and Non-Respondents to a Questionnaire and between Participantsand Non-Participantsin an Examination-	2
20	Google scholar	Michio UEMATSU, Noriko TAKEI, Makiko ITOU, et al.	2000	自己記入式質問紙の有用性に関する口腔診査結果との対比による評価	Evaluation of the Effectiveness of Self-administered Questionnaire by Association with Oral Examination	2
21	Google scholar	Kanto Araki, Yukihiro Sugita and Katsuyoshi Mizukami	2020	行動分析学的フィードバックとマウススプレーを用いた口腔衛生管理による口腔乾燥症に対する有効性	The effectiveness of mouth spray and oral management in patients with xerostomia	2
22	Google scholar	Takako Fujinaka, Shiori Todoko and Kumiko Fukumoto	2006	要介護高齢者のための口腔ケアネットワークの構築 歯科に関する保健・医療・福祉の連携	Establishment of an oral care network for the elderly requiring nursing care Cooperation among health, medical care, and welfare related to dentistry	2
23	Google scholar	Shin ARAI, Masako TATEISHI, Satoshi TERANAKA, et al.	2021	健常者における口腔立体認知―口腔知覚判定研究用キット DF8 を用いた検討―	Oral Stereognosis in Healthy Subjects: A Study Using the DF8 Kit for Oral Sensation Research	2

No.	Database	Author Name	Year	Title JPN	Title ENG	Reason of exclusion
24	Google scholar	Takatsugu MUTOH, Eiki HONDA, Nobuko MAEDA, et al.	2000	長期療養者ならびに寝たきり者の口腔常在微生物叢に関する研究	Oral MicrobialFIora in Institutionalized ElerlyPeople	2
25	Google scholar	Jun AIDA, Kakuhiro FUKAI, Michiko FURUTA, et al.	2017	歯科医院への定期健診はどのような人が受けているのか	Who Receives Regular Dental Checkups? Inequalities in Access to Dental Care: The 8020 Health Study	2
26	Google scholar	MIURA Yoshiko, SHINADA Kayoko, SHIMOYAMA Kazuhiro, et al.	2002	定期的に歯科検診と口腔ケアを受けていた成人の歯の状況	A Study of Oral Status of Adults who Received Regular Checkups and Professional Care	2
27	Google scholar	Sakiko TAKAKU, Etsuko MOTEGI, Mayumi NOMURA, et al.	2012	千葉県生涯大学校に通学する60~70歳代学生の口腔内診査および骨密度調査	Oral status and BUA in elderly persons in sixties and seventies at senior colleges in Chiba Prefecture	2
28	Google scholar	Miehiko TAMURA	2005	成人における口腔健康習慣と口腔保健状況との関連	Relationship between Oral Health Behavior and Oral Health Status in Adults	2
29	Google scholar	Youichirou Uchiyama	2010	ADLが低下した患者における口腔内細菌数の日内変動	Diurnal Variation of Oral Bacterial in Patients with Reduced ADL	2
30	Google scholar	Toshio Moritani, Masahiro Takahashi, Yoshiko Someya et al.	2002	岩手県85歳追跡調査より85歳における口腔内気体のVSCについて：実態と口腔内診査所見との関連	VSC Gases from the Follow-up Survey of 85-Year-Olds in Iwate Prefecture: Relationship between Actual Conditions and Oral Examination Findings	2
31	Google scholar	Koki Shikura, Takaaki Shikkura, Ryutaro Uchikawa et al.	2020	第3次産業勤労者の口腔保健行動と意識	Oral Health Behavior and Attitudes of Tertiary Industry Workers	2
32	Google scholar	Nanami Sawada, Satoko Takeuchi, Ayano Tabata et al	2020	高校生におけるDMF歯数の増加と口腔保健行動との関連	Association between increased number of DMF teeth and oral health behaviors among high school students.	2
33	Google scholar	Katsuya Iijima, Tomoki Tanaka and Aki Kuroda	2015	(虚弱化予防)から要介護状態に至る口腔ケアの包括的対策の構築に関する研究	Research on the comprehensive measures for oral care to the need for nursing care	2
34	EMBASE	Saito M., Shimazaki Y., Fukai K., Furuta M., Aida J., Ando Y., Miyazaki H., Kambara M.	2019		Risk factors for tooth loss in adult Japanese dental patients: 8020 Promotion Foundation Study	2
35	Google Scholar	Nakayama, Yoshimi; Mori, Mitsuru	2012		The relationship between number of natural teeth and oral health behavior in adult Japanese people	2
36	EMBASE	Morita I., Nakagaki H., Toyama A., Hayashi M., Shimozato M., Watanabe T., Tohmatsu S., Igo J.	2000		An oral health scoring system for promoting 8020 achievement in residents	2
37	Google scholar	Noriko Yamada, Fusayo Kobayashi, Akemi Nakazawa, et al.	2018	口腔ケアの方法ー歯磨き調査の結果をもとにー	Considerations on oral care method —Issues found from survey of tooth brushing—	3
38	Google scholar	Miyazaki Haruyo, Motegi Etsuko, Saito Chiaki, et al.	2001	8020達成者の口腔内模型および頭部X線規格写真分析結果について	A study of model and cephalograms in elderly persons over 80 years old with at least20 teeth	3

No.	Database	Author Name	Year	Title JPN	Title ENG	Reason of exclusion
39	Google scholar	Miyazaki Haruyo, Motegi Etsuko, Saito Chiaki, et al.	2004	８０２０達成者の歯科疾患罹患状況および生活と健康に関する調査結果について	Report on dental diseases, quality of life, oral health and general health of elderly persons over 80 years old with at least 20 teeth	3
40	Google scholar	Masaaki YOKOYAMA, Takahito YONEZU, Masaaki YOKOYAMA, et al.	2009	徳島県における妊婦歯科健診受診者の口腔保健の現状および低体重児出産との関連性	Oral Health Status of Pregnant Women Examined in Tokushima Prefecture, and its Relationship with a Low-birth-weight Outcome	3
41	Google scholar	Yoshinori TAKANO, Akira KOMATSUZAKI and Takehiko SUETAKA	1995	口腔内状況が食品摂取におよぼす影響に関する研究	Analysis of Dental Health InfiuencingFood Intake	3
42	Google scholar	Sachiyo ISHIHARA, Tomohiko CHUBACHI, Hirotaka MURAI, et al.	2001	老人病院入院患者の全身疾患と口腔内状況について	Systemic Disease and Oral Condition of Inpatients at a Geriatric Hospital	3
43	Google scholar	Kouko Chida, Keiko Yamada, Noriko Kanou, et al.	2003	中学生の口腔保健に関する意識調査	Survey of Attitude toward Oral Health in Middle School Students	3
44	Google scholar	Akira Komatsuzakit, Akira Ezura, Takehiko Suetaka et al.	2007	介護老人保健施設入所者の食事内容と口腔・全身状況との関連性に関する検討	A study on the relationship between dietary content and oral and systemic conditions of residents in geriatric healthcare facilities	3
45	Google scholar	Rumi Tano, Yoshihide Yabuki, Yoichi Fukuzawa et al.	2010	都市住民における歯科医院への受診状況からみた口腔衛生状態と QOL との関連	Relationship between Oral Hygiene Status and Quality of Life in Urban Residents in terms of Visits to Dental Clinics	3
46	Google scholar	Yutaka Satoh, Toshikazu Yasui and Hiromasa Endoh	1998	埼玉県下における在宅要介護高齢者の口腔保健に関する調査研究 : 第 2 報 口腔内状況と生活環境との関連性について	A Survey Study on Oral Health of Elderly People Requiring Nursing Care at Home in Saitama Prefecture : Second Report The Relationship between Oral Conditions and Living Environment	3
47	Google scholar	Keiji Takekoshi, Junichiro Kotani, Yutaka Ueda et al.	1997	重度痴呆性老人における食事形態および口腔内状況について	Eating Patterns and Oral Conditions in Severely Demented Elderly People	3
48	Google scholar	Yasuko Kanai and Nobuko Mizokawa	1997	老人病院入院患者の口腔内状況と ADL の関係	Relationship between Oral Status and ADL of Inpatients in a Geriatric Hospital	3
49	Google scholar	Ichizo Morita, Hisayoshi Morioka Yoshikazu Abe et al.	2021	高齢者における服薬薬剤成分数と口腔機能低下の関係	Relationship between the Number of Medication Components and Oral Functional Decline in the Elderly	3
50	Google scholar	Chieko Kubota	2014	自立高齢者の栄養状態と口腔状況に関連する因子 : 大学病院歯科外来に受診している高齢患者の分析	Factors Related to Nutritional Status and Oral Status of Independent Elderly People : An Analysis of Elderly Patients Visiting Dental Outpatient Clinic of University Hospital	3
51	Google scholar	Toshiya Kashiwabara, Tetsuo Ichikawa, Naeko Kawamoto et al.	1996	老人病院入院患者の口腔状態とデンチャープラークの細菌構成について	Oral Status and Bacterial Composition of Denture Plaque in Patients Admitted to a Geriatric Hospital	3
52	Google scholar	Barkhwa Kim, Synichi Oka and Koji Takada	2006	口腔外科手術予定の中高齢患者における歯の残存状況と血管壁硬度の関係について	Relationship between tooth residual status and vascular wall hardness in middle-aged and elderly patients scheduled for oral surgery.	3

No.	Database	Author Name	Year	Title JPN	Title ENG	Reason of exclusion
53	Google scholar	Kazuhiro Shimoyama, Osamu Ooashi, Masahiro Umino et al.	1992	高齢者の QOL と抑うつ感に対する口腔内状況の影響	Effect of Oral Status on Quality of Life and Depression in the Elderly	3
54	Google scholar	Toshiki Moriya, Masahiro Takahashi, Tamotsu Sato et al.	2005	85 歳における口腔内気体の VSC およびその関連口腔内診査所見と ADL および QOL の測定指標との関連について	Association of VSC Oral Gases and Related Oral Examination Findings with ADL and Quality of Life Measures in 85-Year-Olds	3
55	Google scholar	Chiho Nakajima, Satoko Takeuchi, Daisuke Eguni et al.	2020	福祉施設利用高齢者における日常生活自立度と口腔機能との関係	Relationship between the level of independence in daily living and oral function among elderly patients in welfare facilities.	3
56	Ichushi	Yukumi Kanehira ,Korenori Arai, Koji Kawasaki et al.	2014	喪失歯数に対するフッ化物配合歯磨剤の影響(Effect of fluoridated dentifrice on the number of missing teeth)(英語)	Effect of fluoridated dentifrice on the number of missing teeth	3
57	Ichushi	Katsuhiko TAURA, Jun AIDA, Yuichi ANDO, et al.	2010	フッ化物洗口の都道府県別にみた普及の推移 国の政策が果たした役割の検討	Change in Conditions Affecting the Regional Spread of School-based Fluoride Mouth Rinsing : Study on the Role of National Policy	3
58	Ichushi	Kazunari KIMOTO, Katsuhiko TAURA, Chieko TAGUCHI, et al.	2009	日本における集団応用でのフッ化物洗口に関する実態調査 施設別、都道府県別の普及状況(2008)	National Survey of School-based Fluoride Mouth Rinsing Program in Japan : Regional Spread to Various Kinds of Schools in 2008	3
59	Ichushi	Yukio KANEKO, Etsuko MOTEGI, Takao YAMAGUCHI, et al.	2007	8020 達成者の歯にみられた cervical lesion について	Cervical lesions in elderly persons over 80 years old with more than 20 teeth	3
60	Ichushi	Yuichi ANDO, Yukio HIRATA, Seiko ISHIKAWA, et al.	2006	地方自治体におけるフッ化物利用に関する施策の現状	The Current Status of Health Policies for Fluoride Use at the Level of Local Government in Japan	3
61	Ichushi	SHIGERU YANAGISAWA, MITAUO YAMAGISHI, TAKESHI YOKOYAMA, et al.	2006	長野県の歯科医療機関におけるフッ化物応用の実施状況について	The status of fluoride application programs at dental clincs in Nagano Prefecture	3
62	Ichushi	Akihiro YOSHIHARA, Syojiro KATAOKA, Kaname NONO, et al.	2005	公診連携を目指した 8020 育成事業の評価	The Evaluation of the "8020 Improvement Project" by the Cooperation between Public Schools and Dental Clinics	3
63	Ichushi	Juichi MIYAMURA, Yasushi YODA, Ichiro SAKAMOTO, et al.	1998	残存歯 20 本以上を有する高齢者の顎関節症候の実態調査	A study on temporomandibular join't symptoms and signs of elderly with more than 20 teeth	3
64	Ichushi	Kaoru BANDOH, Takeyoshi KOSEKI, Eiichi SUGIYAMA, et al.	1995	歯周治療の長期観察について	Long Term Observations of Periodontal Therapy	3
65	EMBASE	Saito M., Shimazaki Y., Fukai K., Furuta M., Aida J., Ando Y., Miyazaki H., Kambara M.	2020		A multilevel analysis of the importance of oral health instructions for preventing tooth loss: The 8020 Promotion Foundation Study of Japanese Dental Patients	3
66	Google Scholar	Ueda, Hiroshi;	2013		Can the Number of Present Teeth Be a Succinct Indicator of Diseases among the Elderly?: Implications from the Hyogo 8020 Survey	3

No.	Database	Author Name	Year	Title JPN	Title ENG	Reason of exclusion
67	Google Scholar	Morita, Kentaro; Shinohara, Ryoji; Sugisawa, Yuka; Sawada, Yuko; Tanaka, Emiko; Hirano, Maki; Watanabe, Taeko; Onda, Yoko; Kawashima, Yuri; Ito, Sumio	2011		Oral health behaviors and mortality in a 6-year cohort study of community-dwelling elderly people	3
68	EMBASE	Rodis O.M.M., Shimono T., Matsumura S., Hatomoto K., Matsuo K., Kariya N., Okazaki Y., Ji Y.	2006		Cariogenic bacteria and caries risk in elderly Japanese aged 80 and older with at least 20 teeth	3
69	EMBASE	Shimizu T., Motegi E., Nomura M., Kaneko Y., Takeuchi F., Yamaguchi T., Miyazaki H., Harazaki M., Hirai M., Kurihara S., Yamaguchi H.	2006		A study of occlusion in elderly Japanese over 80 years with at least 20 teeth	3
70	Google scholar	Kumiko MINAGAWA, Akihiro YOSHIHARA, Misuzu SATO, et al.	2018	一般地域住民における主観的な歯や歯肉の健康状態と全身健康状態との関連	Relationship between Self-rated Tooth and Gingival Health and Self-rated General Health among General Japanese Subjects: The 8020 Health Study	4
71	Google scholar	Kakuhiro FUKAI	1998	わが国の成人集団における口腔保健の認知度および歯科医療の受容度に関する統計的解析	Oral health awareness and acceptance of dental care in the Japanese adult population	4
72	Google scholar	Kakuhiro FUKAI, Yoshinobu MAKI and Yoshinori TAKAESU	1996	成人の口腔保健行動とその年齢特性	Oral Health Behavior of Adults in Relationto Age Groups	4
73	Google scholar	Yoshihiko HIROTANI, Sho Kawasu, Jyunji Mukai et al.	2019	地域住民を対象とした口腔ケアチェックとアンケート調査による口腔内環境の現状と悪化因子の検討	Oral Conditions and Aggravating factors - Oral Examination and Questionnaire Survey among Community Dwelling Population -	4
74	Google scholar	Satoshi Harusakku, Akihito Tsutsui, Kenji Sakai et al	1996	職域における口腔健康教育の効果および歯科保健意識と行動変容因子との関連性の検討	Effect of Oral Health Education at Occupational Areas, and Behavioral Changes	4
75	Google scholar	Masahiko Taura, Kazunari Motoki, Yasuhisa Arakawa et al.	2006	日本の小学校における保健学習用の口腔関連記述に関する検討	Oral Health Education at Elementary School in Japan	4
76	Google scholar	Shin, Okumura, Tatsuro Miyake, Masaki Kanbara	2003	口腔年齢指標による地域口腔保健評価	Community Oral Health Evaluation by Oral Age Index	4
77	Google scholar	Aya Iwamoto, Yuko Ishikara, Minoru Yagi et al	2012	リスク発見・保健指導重視型の成人歯科健診プログラムにおける口腔保健行動の変化	Changes of Oral Health Behaviors by Health Instruction Oriented Adults Oral Health Checkup Programs	4
78	Google scholar	Yoshie Usui, Hiroko Miura and Yoh Tamaki	2013	超高齢社会における歯科口腔保健の今後のニーズと課題に	Perception of Dental Professionals about Needs for Oral Health in Super-ageing Society	4

No.	Database	Author Name	Year	Title JPN	Title ENG	Reason of exclusion
				関する歯科有識者への意識調査		
79	Google scholar	Mirai Fukuda, Ayami Sato, Yoko Uchida	2018	自宅で生活する認知症高齢者の認知機能重症度別にみた口腔清掃自立度の特徴 ～IADL、ADL との比較～	Characteristics of Independency Oral Health Care among Cognitive Compromized Community dwelling People - IADL and ADL -	4
80	Google scholar	Ayako Sakoda, Michiko Konishi	2002	在宅療養を支援する介護者の口腔ケア行動とその要因に関する研究	A Study on Oral Care Behavior and Related Factors among Caregivers who Support Home Care	4
81	Google scholar	Masami Murano, Youko Kawaguchi, Kayoko Shinada et al.	1999	某官庁職員の口腔内保健状況および保健行動に関する研究	A Study on Oral Health Status and Health Behavior of Government Employees	4
82	Google scholar	Atsushi Umetsu and Mutsuko Mihashi	2017	口腔ケア時の洗浄液の飛散状況および口腔環境調査	Mouthwash splashing during Oral Care and Oral Condition	4
83	Google scholar	Kunihiko Terashita, Fumiaki Shinsho, Masatoshi Fujita et al.	1997	成人および高齢者の歯科健康診査結果にみる口腔保健の実状	The Actual State of Oral Health in the Results of Dental Health Examinations for Adults and the Elderly	4
84	Google scholar	Uku Pekujun	2012	高齢者における口腔状態と全身健康が QOL に及ぼす影響：韓日比較利用統	Effects of Oral Status and General Health on Quality of Life among the Elderly: A Korean-Japanese Comparative Study	4
85	Google scholar	Junko Akamatsu, Mitsuo Kishi, Akiko Abe et al.	2015	岩手医科大学附属病院の医科歯科連携における口腔ケア外来の役割と課題	The Role of Outpatient Oral Care in Medical and Dental Collaboration at Iwate Medical University Hospital	4
86	Google scholar	Toru Ichihashi, Norihide Nisinoue, Yasuji Takada et al.	2013	任意参加方式の職域歯科保健活動への参加者と不参加者における口腔内状態および保健行動の比較	Comparison of Oral Health Status and Health Behaviors between Participants and Non-participants in a Voluntary Community Dental Health Activity	4
87	Google scholar	Mitsuhiro Amazakia and Chihiro Kemuriyama	2018	Health Action Process Approach を用いた大学生の口腔保健行動の検討	An Examination of Oral Health Behaviors of College Students Using the Health Action Process Approach	4
88	Google scholar	Rika Shibuya, Yui Okazawa, Daisuke Hinode et al.	2016	幼児のフッ化物配合歯磨剤利用に関する口腔保健行動	Oral Health Behaviors of Young Children Using Fluoride Toothpaste	4
89	Google scholar	Noriko Takei, Atsushi Fujimoto, Emiko Kimoto et al.	2009	高齢者の口腔機能の評価と管理のシステム化に関する研究	A Study on the Systematization of Evaluation and Management of Oral Function in the Elderly	4
90	Google scholar	Hiroshi Narumi and Hideko Shimodate	2013	本校の歯科口腔保健の推進に対する取り組みと成果～平成 22 年度準県一、平成 23 年度県一～	Efforts and Achievements in the Promotion of Oral Health at School - Ranked the First Position1 in 2010 and 2011 in the prefecture-	4
91	Google scholar	Atsuko Kawamura, Yuki Shinoki, Hikari Tamura et al.	2020	地域高齢者の口腔機能の実態調査と摂食機能の健康管理に関する意識の変化	A Survey of Oral Function and Changes in Attitudes toward Health Management of Eating Function among Community-Dwelling Elderly People	4

No.	Database	Author Name	Year	Title JPN	Title ENG	Reason of exclusion
92	Google scholar	Yoshiyuki Hagiwara, Tomoko Morino, Mitsuko Seki et al.	2012	介護老人福祉施設における口腔ケアの実態	Oral Health Care in Long-term Care Facilities for the Elderly	4
93	Google scholar	Masao Ishikawa, Noriko Takei, Takanori Ishii et al.	2015	グループホームにおける口腔機能向上プログラム介入による 認知機能の低下抑制効果について	Oral Function Improvement Program in Group Homes for Preventing Decline in Cognitive Function	4
94	Google scholar	Michiko Honma, Kyoko Koiso, Chizuru Seki et al.	2016	新潟県の介護保険施設入所高齢者への口腔健康管理に関する医療福祉 4 専門職種の認識と励行度の比較	A Comparison of the Perceptions and Encouragement of Four Medical and Welfare Professionals Regarding Oral Health Care for Elderly Residents of Long-term Care Facilities in Niigata Prefecture	4
95	Google scholar	Takashi Maruyama	2021	S 市における通所介護利用者の口腔機能の向上のための方策に関する研究-通所介護と歯科診療所の連携実態の調査結果から	A Study on the Measures to Improve the Oral Functions among People who Utilize Day-care Center in S City: Results of a Survey on the Collaboration between Day-care Centers and Dental Clinics	4
96	Google scholar	Tsuneyuki Kuratate, Masaaki Iwaku	2002	小中学校新学習指導要領（文部科学省）における口腔衛生教育の実践的な研究	Practical Research on Oral Hygiene Education in the New Curriculum Guidelines for Elementary and Junior High Schools	4
97	Google scholar	Atsushi Ooyama, Yuichi Ando, Manabu Morita	2015	糖尿病と口腔保健アセスメント項目の関連性の検討 : 生活歯援プログラムを利用して	Examining the Relationship between Diabetes Mellitus and Oral Health Assessment Items: Utilizing the Lifestyle Dental Assistance Program	4
98	Google scholar	Noriko Kumagai, Jyuzo Morita, Haruo Nakagaki et al.	2005	“歯の健康づくり得点”による住民の歯の喪失予測性評価	"Oral Healthiness Score for 8020" Predicts Loss of Teeth in Village Residents	4
99	Google scholar	Kazunori Ikebe, Hidekazu Namba, Nozomu Tanioka et al.	1998	自立している高齢者の口腔と全身の健康	Oral and systemic health of independent older adults.	4
100	Ichushi	Fumiko OKADA, Shoichi YANO, Hideki HAYASHI et al.	2019	九州地区で口腔インプラント治療に携わる歯科医師および歯科衛生士の歯と歯周組織の状態についての研究	Dental and Periodonatal Status of Dentists and Dental Hygienists Engaging in Oral Implant Treatments in the Kyushu Area	
101	Ichushi	Yonesato Hirata, Naohiko Kinoshita, Michiko Honma et al.	2018	初診時における咬合崩壊予測に関する研究 歯科診療所の経年データを用いた Cox 比例ハザードモデル	Prediction of Bite Collapse by Proportional Hazard Model, Using Patient Dental Records	4
102	Ichushi	Tomoko Imai, Noboru KItagawa, Yuji Sato et al.	2011	補綴治療が無歯顎者の咀嚼機能に与える影響	The Effects of Dental Prosthodontic Treatments on Masticatory Function in the Edentulous Ptients	4
103	Ichushi	Masashi SUGISAKI, Naohisa TAKANO, Katsuhiko HAYASHI et al.	2011	東京都内一般歯科診療所受診者における顎関節症スクリーニングと性別就業内容に関する予備研究	A pilot study on the relationship between descriptions of job contents by gender and screening for temporomandibular disorders in dental patients at general clinical offices in Metropolitan Tokyo using a questionnaire	4
104	Ichushi	Satoru HARESAKU, Akihito TSUTSUI, Kenji SAKAI, et al.	2004	職域における口腔健康教育の効果及び歯科保健意識と行動変容因子との関連性の検討	Relationships of Dental Factors for Behavior Change with the Effects of an Intervention Program in the Workplace and Consciousness of the Oral Health of Workers	4

No.	Database	Author Name	Year	Title JPN	Title ENG	Reason of exclusion
105	Ichushi	Ichizo MORITA, Atsushi TOYAMA, Noriko KUMAGAI, et al.	2003	インターネット調査による歯科に関する用語の認知と個人属性の関係	Personal Attributes and the Degree of Understanding of Dental Terms as Assessed by Internet Survey	4
106	Ichushi	Tomohiro ABE	1998	80 歳以上の高齢者における歯科保健に関する研究 山形県上山市での調査	A Study of the Oral Health of Elderly Persons over 80 Years of Age : The Investigation of Kaminoyama City in Yamagata Prefecture	4
107	PubMed	Haruyo Miyazaki, Etsuko Motegi, Kenichi Yatabe, Hideharu Yamaguchi, Yoshinobu Maki	2005		Cephalometric study of elderly with nearly intact dental arches.	4
108	Google scholar	Atsushi Minami, Kazuhiro Nakata, Hideki Okuyama, et al.	2004	介護保険施設と歯科医療施設の連携による口腔機能改善への取り組みとその評価	An Assessment of Functional Oral Rehabilitation Program Provided by Dentists and Dental Hygienists for Institutionalized Dependent Elderly People	5
109	Ichushi	Hitoshi OSADA, Keiko SHIINA and Yuichi ANDO	2017	大都市居住 80 歳高齢者の現在歯保有状況に関する記述疫学的研究 2012 年の東京都杉並区の調査から	Tooth Retention in Metropolitan City Residents Aged 80 Years in 2012: A Descriptive Epidemiological Study	5
110	Ichushi	Haruko Sekiguchi, Kuniko Kurabayashi, Hiromi Sato, et al.	2007	通所施設での円滑な口腔機能向上サービスを目指して モデル事業から見えてきたこと	Toward Smooth Oral Function Improvement Services at Day Care Facilities: Findings from a Model Project	5
111	Google scholar	Zhu Min	2017	「8020 運動」 と社会疫学からみた口腔ケア	Oral care from the perspective of the "8020 Movement" and social epidemiology	6
112	Google scholar	Kentaro Morita	2013	口腔保健行動と生命予後に関する経年的研究	Longitudinal Study of Oral Health Behaviors and Life Expectancy	6
113	Google scholar	Yoko Hasegawa, Ayumi Sadakane, Hiromitsu Kishimoto, et al.	2018	口腔機能低下は高齢者の転倒リスクを高めるのか?(原著論文)	Does Oral Dysfunction Increase the Risk of Falls in the Elderly? (Original Research Paper)	6
114	Google scholar	Hiromitsu Kishimoto, Nobuhide Horii, Takashi Sawada, et al.	2018	丹波篠山圏域在住高齢者における QOL と口腔機能との関連性(原著論文)	Relationship between Quality of Life and Oral Functioning in the Elderly Living in the Tamba-Sasayama Area (Original Research Paper)	6
115	Google scholar	Masaya Nishikawa, Satoru Yamaguchi, Eiji Nakamichi, et al.	2019	肺がん患者への周術期における口腔機能管理の有用性に関する研究(原著論文)	Study on the usefulness of perioperative oral function management for lung cancer patients (Original article)	6
116	Google scholar	Takuya Kobayashi, Masashi Hatamoto, Ayaka Nakasato	2021	口腔機能の改善が脳認知機能を向上させる(原著論文)	Improvement of Oral Function Improves Brain Cognitive Function (Original article)	6
117	Google scholar	Hiroyuki Suzuki, Chiaki Matsubara, Rena Hidaka, et al.	2019	多職種連携医療(NST)における栄養と口腔機能の関連と歯科介入効果の検討(原著論文)	The relationship between nutrition and oral function and the effect of dental intervention in multidisciplinary collaborative healthcare (NST) (Original article)	6
118	Google scholar	Okinori Shikura	2020	第 3 次産業勤労者の口腔保健行動と意識	Oral health behavior and awareness of tertiary industrial workers	6

1= not interested exposure, 2=not interested outcome, 3=no comparison or control group, 4=irrelevant, 5=lacking necessary information, 6=not peered review journal

Risk of Bias Assessment

Table S2. Risk of Bias Assessment of Cross-sectional/Cohort Studies(5)

Study	Assessment Questions														
	1	2	3	4	5	6	7	8	9	10	11	12	13	14	Overall
Motegi et al.,(30)	+	+	?	-	-	-	-	x	-	-	+	?	-	-	3
Ohazama et al.,(29)	+	+	?	-	-	-	-	x	-	-	+	?	-	-	3
Hashimoto et al.,(7)	+	+	?	+	-	-	-	x	+	-	+	?	-	-	5
Yamaga et al.,(9)	+	+	?	+	-	-	-	+	+	-	+	?	-	+	7
Hashimoto et al.,(8)	+	+	?	-	-	-	-	x	+	-	+	?	-	-	4
Nakane et al.,(10)	+	+	-	+	-	+	+	+	-	+	-	?	-	-	7
Kanda et al.,(11)	+	+	?	+	-	+	-	+	-	-	+	+	-	+	8
Ansai et al.,(12)	+	+	+	+	-	-	-	+	-	-	+	x	-	-	6
Hirano et al.,(13)	+	+	?	+	-	-	-	+	+	-	+	?	-	+	7
Matsui et al.,(14)	+	+	?	+	-	-	-	+	-	-	+	?	-	+	6
Sato et al.,(15)	+	+	?	+	-	-	-	x	+	-	+	?	-	-	5
Yamamoto et al.,(16)	+	+	?	+	-	-	-	+	+	-	+	?	-	-	6
Takemae(17)	+	+	+	+	-	-	-	+	+	-	+	?	-	-	7
Matsuo et al.,(18)	+	+	?	+	-	-	-	+	+	-	+	?	-	-	6
Wakikawa et al.,(19)	+	+	?	+	-	-	-	+	+	-	+	?	-	+	7
Shikura et al.,(21)	+	+	+	+	-	-	-	+	-	-	-	?	-	+	6
Takeuchi et al.,(28)	+	+	?	-	-	-	-	+	+	-	+	x	-	-	5
Morita et al.,(22)	+	+	?	+	-	-	-	+	+	-	+	x	-	?	6
Morita et al.,(23)	+	+	?	+	-	+	+	x	+	+	+	x	?	+	9
Iwasaki et al.,(6)	+	+	+	+	-	+	+	+	+	+	+	?	?	+	11
8020 promotion foundation(24)	+	+	+	+	-	-	-	+	-	-	+	x	-	-	6
8020 promotion foundation(25)	+	+	+	+	-	+	-	+	+	-	+	x	-	-	8
8020 promotion foundation(26)	+	+	?	+	-	+	-	x	+	+	+	x	-	-	7
8020 promotion foundation(27)	+	+	-	+	-	-	-	+	-	-	+	x	-	+	6

+ Yes/Low risk - No/High risk x Other (Not applicable) ? Not mentioned

Table S3. Risk of Bias Assessment Question for Cross-sectional/Cohort Studies(5)

No	Question
1	Was the research question or objective in this paper clearly stated?
2	Was the study population clearly specified and defined?
3	Was the participation rate of eligible persons at least 50%?
4	Were all the subjects selected or recruited from the same or similar populations (including the same time period)? Were inclusion and exclusion criteria for being in the study prespecified and applied uniformly to all participants?
5	Was a sample size justification, power description, or variance and effect estimates provided?
6	For the analyses in this paper, were the exposure(s) of interest measured prior to the outcome(s) being measured?
7	Was the timeframe sufficient so that one could reasonably expect to see an association between exposure and outcome if it existed?
8	For exposures that can vary in amount or level, did the study examine different levels of the exposure as related to the outcome (e.g., categories of exposure, or exposure measured as continuous variable)?
9	Were the exposure measures (independent variables) clearly defined, valid, reliable, and implemented consistently across all study participants?
10	Was the exposure(s) assessed more than once over time?
11	Were the outcome measures (dependent variables) clearly defined, valid, reliable, and implemented consistently across all study participants?
12	Were the outcome assessors blinded to the exposure status of participants?
13	Was loss to follow-up after baseline 20% or less?
14	Were key potential confounding variables measured and adjusted statistically for their impact on the relationship between exposure(s) and outcome(s)?

Table S4. Risk of Bias Assessment of Intervention Studies(31)

Study	Assessment Questions														
	1	2	3	4	5	6	7	8	9	10	11	12	13	14	Overall
Shikura et al.,(21)	+	?	-	-	-	+	-	+	?	?	+	-	+	x	5

+ Yes/Low risk	- No/High risk	x Other (Not applicable)	? Not mentioned

Table S5. Risk of Bias Assessment Question for Cross-sectional/Cohort Studies(31)

No	Question
1	Was the study described as randomized, a randomized trial, a randomized clinical trial, or an RCT?
2	Was the method of randomization adequate (i.e., use of randomly generated assignment?
3	Was the treatment allocation concealed (so that assignments could not be predicted)?
4	Were study participants and providers blinded to treatment group assignment?
5	Were the people assessing the outcome blinded to the participants' group assignments?
6	Were the group similar at baseline on important characteristics that could affect outcome (e.g., demographics, risk factors, co-morbid conditions)?
7	Was the overall dropout rate from the study at its endpoint 20% or less than the number originally allocated to treatment?
8	Was the differential drop-out rate between groups at the study's endpoint 15% or less?
9	Was there high adherence to the intervention protocols for each treatment group?
10	Were other interventions avoided or similar in the groups (e.g., similar background treatments)?
11	Were outcomes assessed using valid and reliable measures, implemented consistently across all study participants?
12	Did the authors report that the sample size was sufficiently large to be able to detect a difference in the main outcome between groups with at least 80% power?
13	Were outcomes reported or subgroups analyzed prespecified (i.e., identified before analyses were conducted)?
14	Were all randomized participants analyzed in the group to which they were originally assigned (i.e, did they use an intention-to treat analysis)?

Table S6. Risk of Bias Assessment for Intervention Studies(31)

Study	Assessment Questions												
	1	2	3	4	5	6	7	8	9	10	11	12	Overall
Ibayashi	+	-	-	-	-	+	+	x	-	+	+	x	5

+	Yes/Low risk	-	No/High risk	x	Other (Not applicable)	?	Not mentioned

Table S7. Risk of Bias Assessment Question for Intervention Studies (31)

No	Question
1	Was the study question or objective clearly stated?
2	Were eligibility/selection criteria for the study population prespecified and clearly described?
3	Were the participants in the study representative of those who would be eligible for the test/service/intervention in the general or clinical population of interest?
4	Were all eligible participants that met the prespecified entry criteria enrolled?
5	Was the sample size sufficiently large to provide confidence in the findings?
6	Was the test/service/intervention clearly described and delivered consistently across the study population?
7	Were the outcome measures prespecified, clearly defined, valid, reliable, and assessed consistently across all study participants?
8	Were the people assessing the outcomes blinded to the participants' exposures/interventions?
9	Was the loss to follow-up after baseline 20% or less? Were those lost to follow-up accounted for in the analysis?
10	Did the statistical methods examine changes in outcome measures from before to after the intervention? Were statistical tests done that provided p values for the pre-to-post changes?
11	Were outcome measures of interest taken multiple times before the intervention and multiple times after the intervention (i.e., did they use an interrupted time-series design)?
12	If the intervention was conducted at a group level (e.g., a whole hospital, a community, etc.) did the statistical analysis take into account the use of individual-level data to determine effects at the group level?

REFERENCES

1. Iwashita H, Tsukiyama Y, Kori H, Kuwatsuru R, Yamasaki Y, Koyano K. Comparative cross-sectional study of masticatory performance and mastication predominance for patients with missing posterior teeth. J Prosthodont Res. 2014;58(4):223-9.
2. Rana B, Harkirat S, Carlos Q, Sonica S. Impact of Poor Oral Health on Community-Dwelling Seniors: A Scoping Review:. Health Services Insights. 2021;14:19.
3. National Heart, Lung, and Blood Institute. Managing Overweight and Obesity in Adults: Systematic Evidence Review from the Obesity Expert Panel 2013 [Available from: https://www.nhlbi.nih.gov/health-topics/managing-overweight-obesity-in-adults.
4. Guyatt GH, Oxman AD, Montori V, Vist G, Kunz R, Brozek J, et al. GRADE guidelines: 5. Rating the quality of evidence--publication bias. J Clin Epidemiol. 2011;64(12):1277-82.
5. National Institutes of Health. Quality Assessment Tool for Observational Cohort and Cross-Sectional Studies 2014 [Available from: https://www.nhlbi.nih.gov/health-pro/guidelines/in-develop/cardiovascular-risk-reduction/tools/cohort.
6. Iwasaki M, Sato M, Minagawa K, Ansai T, Ogawa H, Yoshihara A. Correlation between Number of Teeth and Mortality: The Significance of Retaining 28 Teeth during Later Life. Journal of Dental Health. 2019;69(3):131-8.
7. Hashimoto M, Yamanaka K, Shimosato T, Ozawa A, Takigawa T, Hidaka S, et al. Oral condition and health status of elderly 8020 achievers in Aichi Prefecture. Bull Tokyo Dent Coll. 2006;47(2):37-43.
8. Hashimoto M, Yamanaka K, Shimosato T, Furuzawa H, Tanaka H, Kato H, et al. Oral condition and health status of people aged 80-85 years. Geriatrics & Gerontology International. 2006;6(1):5.
9. Yamaga T, Yoshihara A, Ando Y, Yoshitake Y, Kimura Y, Shimada M, et al. Relationship between dental occlusion and physical fitness in an elderly population. J Gerontol A Biol Sci Med Sci. 2002;57(9):M616-20.
10. Nakane S, Hiraiwa K, Osawa R, Osawa M, Hatori K. A Study to Evaluate the Effectiveness of Using the “Passport to Healthy Teeth”, a 8020 Project to Protect and Nurture the First Permanent Molar. Journal of Dental Health. 2013;63(1):28-34.
11. Kanda M, Ueda H, Hashimoto T. A Three-year Follow-up Study of the Relationship among the Numbers of Present Teeth, the Loss of Teeth and Medical Expenditure in the Elderly. Japanese Journal of Gerodontology. 2008;23(2):132-9.
12. Ansai T, Hamasaki T, Awano S, Akifusa S, Katoh Y, Arimoto T, et al. Relationship between Oral Health and Exercise Function of Elderly Persons 80 Years Old in Fukuoka Prefecture. Journal of Dental Health. 2000;50(5):783-9.
13. Hirano H, Ishiyama N, Watanabe I, Suzuki T. Oral Examination of Longitudinal Interdisciplinary Study on Aging (II) Masticatory ability in relation to oral status and general health. Japanese Journal of Gerodontology. 1993;7(2):150-6.
14. Matsui T, Takemae Y, Tamura H, Omine T, Sone F. The Relationship between the Amenity and Psychological or Physical State in Oral Cavity in the Aged. Journal of the Kyorin Medical Society. 1995;26(3):379-86.
15. Sato M, Kitagawa N, Sato Y, Utanohara H, Asakura T. Investigation of Advantages and Problems in 8020 Achievers. Japanese Journal of Gerodontology. 2007;22(1):12-24.
16. Yamamoto T, Shimabara S, Ariyoshi Y, Kimura Y, Yamaga T, Kouno K. Clinical Study on Oral Factors Influencing Salivary Secretion -About Adults. Journal of Japanese Society for Evidence and the Dental Professional. 2011;3(1):42-8.
17. Takemae T. A study on the Quality of Life among the Middle or Advanced Aged -An Approach from the point of Oral Hygiene-. Journal of the Kyorin Medical Society. 1996;27(3):197-213.

18. Matsuo K, Taniguchi H, Nakagawa K, Kaznazawa M, Furuya J. Relationships between Deterioration of Oral Functions and Nutritional Status in Elderly Patients in an Acute Hospital. Japanese Journal of Gerodontology. 2016;31(2):123-33.
19. Wakikawa T, Nishihira A, Fujita J, Sendo W, Matsui N, Shimatan H. An investigation into the intraoral sanitary conditions in hemodialysis patients and evaluation of the relationship between remaining teeth and laboratory values associated with nutritional status. The Journal of Japanese Association of Dialysis Physicians. 2013;46(6):535-43.
20. Ibayashi H, Yano J, Pham T, Minh, Tanaka M, Nishiyama T, Sakai K, et al. Effect of Oral Health Care Program on the Oral Function of the Elderly. Journal of University of Occupational and Environmental Health. 2006;28(4):411-20.
21. Shikura O, Shikura T, Uchikawa R, Yamamoto A, Tomida M. Oral Health Behavior and Awareness of Tertiary Industry Workers: A 2016 Questionnaire Survey in Nishi-ku, Sakai-shi, Osaka. The Japanese Journal of Conservative Dentistry. 2020;63(5):385-95.
22. Morita I, Nakagaki H, Murakami T, Kato K, Mizuno T, Tsuboi S, et al. Nutrition and Diet of 80-Year-Old Japanese Persons Retaining 20 Teeth Per Individual (8020). Journal of Dental Health. 1996;46(3):241-7.
23. Morita M, Kajiura S, Jim-boom K, Okazaki M, Nishigawa M, Hiraiwa M, et al. Effect of Professional tooth Cleaning on Prolongation of Tooth Life. Journal of Dental Health. 1995;45(2):190-5.
24. 8020 Promotion Foundation. National Adult Dental Health Survey Report: 8020 promotion foundation; 2007 [1-124]. Available from: https://www.8020zaidan.or.jp/pdf/jigyo/zenkoku_seijin.pdf.
25. 8020 promotion foundation. 2014-2019 Research Project "Study on the Effectiveness of Dental Medicine to Promote Health" Report: *Relationship between oral (number of teeth, periodontal status) and general health (NCDs, subjective health status):* 8020 promotion foundation; 2020 [23-31]. Available from: https://www.8020zaidan.or.jp/pdf/Dentistry_Enhancement_Effect_2014-2019.pdf.
26. 8020 promotion foundation. 2014-2019 Research Project "Study on the Effectiveness of Dental Medicine to Promote Health" Report: *Results of a survey of the general local population:* 8020 promotion foundation; 2020 [32-9]. Available from: https://www.8020zaidan.or.jp/pdf/Dentistry_Enhancement_Effect_2014-2019.pdf.
27. 8020 Promotion Foundation. 2014-2019 Research Project "Study on the Effectiveness of Dental Medicine to Promote Health" Report: *Understanding the patterns of dental patients visits and the factors that influence them:* 8020 Promotion Foundation; 2020 [40-8]. Available from: https://www.8020zaidan.or.jp/pdf/Dentistry_Enhancement_Effect_2014-2019.pdf.
28. Takeuchi F, Miyazaki H, Nomura M, Motegi E, Harazaki M, Yatabe K, et al. Report on occlusal conditions and occlusal force measured with a pressure sensitive film in elderly persons over 80 years old with at least 20 teeth. The Journal of the Tokyo Dental College Society. 2005;105(2):154-62.
29. Ohazama J, Motegi E, Nomura M, Miyazaki H, Takane Y, Harazaki M, et al. Oral flora in independent over 80-year-olds with more than 20 teeth. Bull Tokyo Dent Coll. 2006;47(1):1-4.
30. Motegi E, Nomura M, Tachiki C, Miyazaki H, Takeuchi F, Takaku S, et al. Occlusal force in people in their sixties attending college for elderly. Bull Tokyo Dent Coll. 2009;50(3):135-40.
31. National Institutes of Health. Assessing Cardiovascular Risk. United States of America: National Heart, Lung, and Blood Institutes; 2013.
